# Risk assessment in clinical trials: it don’t mean an ethical thing if it ain’t got that probability ring!

**DOI:** 10.3332/ecancer.2014.ed42

**Published:** 2014-09-03

**Authors:** T Patrick Hill

**Affiliations:** Senior Policy Fellow, Edward J. Bloustein School of Planning and Public Policy, Rutgers, the State University of New Jersey, USA

Since clinical trials are conducted in a context of inherent uncertainty, the assessment of risk is central to their scientific design and ethical conduct. Substantial evidence points to a pervasive misunderstanding of the concept of risk within the clinical research community that results in risk assessment being one of the least satisfactory parts of trial protocols. At its core, the problem rests with the fact that medicine, unlike the social sciences, economics, and philosophy which now pursue their respective scientific methodologies grounded in a probabilistic paradigm, remains wedded to a deterministic paradigm. However, it is from within the probabilistic rather than the deterministic paradigm that justification for conducting clinical research is to be found.

Since prospectively their outcomes, including benefit and harm, are unknowable, clinical trials take place under conditions of uncertainty. In recognizing this, the Belmont Report (Belmont) [1] called for the assessment of risk involved in participating in clinical trials with three goals in mind. The first, for clinical researchers, is to determine whether the research design poses minimal risk of harm to participants. The second goal, for prospective participants, is to help them to decide whether to participate in the trial. The third goal, for institutional review boards (IRB’s), is to enable members to determine whether the risk of harm relative to the likelihood of benefit favors benefit over harm for trial participants. By way of confirmation, the National Bioethics Advisory Commission (NBAC) declared that “risk is a central organizing principle, a filter through which protocols must pass” [2] if scientific validity, patient participation, and IRB approval are to be considered reasonable.

But given the centrality of risk assessment to scientific validity, a patient’s informed consent to participation and IRB approval, why in a 2002 study would up to two-thirds of respondents find that trial protocols provide insufficient information regarding the likelihood of both risks and benefits [3]? Why is it that so few protocols pass through the NBAC filter and in the way envisaged by Belmont?

Closer reading of Belmont also reveals that risk assessment requires a specific understanding of the concept of risk which:

refers to a possibility that harm may occur. However, when expressions such as ‘small risk’ or ‘high risk’ are used, they usually refer (often ambiguously) both to the chance (probability) of experiencing harm and the severity (magnitude) of the envisioned harm [1, pg7].

It is clear from this language that the term risk is not to be understood as a univocal term, synonymous with the term harm. Yet this is universally the understanding in clinical research protocols. Compounding the misunderstanding is the universally used phrase ‘risks and benefits’ which implies that ‘risk’ is the opposite of ‘benefit’, consequently disposing to no consideration of probability regarding both, even though this is precisely what Belmont requires:

The term ‘benefit’ is used in the research context to refer to something of positive value related to health or welfare. Unlike ‘risk’, ‘benefit’ is not a term that expresses probabilities. Risk [of harms] is properly contrasted to probability of benefits, and benefits are properly contrasted with harms rather than risk of harms. Accordingly, so-called risk/benefit assessments are concerned with the probabilities and magnitude of possible harms and anticipated benefits [1, pg7].

Belmont also requires an assessment of the probability of harms occurring relative to the probability of benefits occurring. But the language is ambivalent, calling for a determination of “the nature, probability and magnitude of risk” [1], with probability distinguished from risk. The ambivalence goes further with subsequent language: “It should also be determined whether an investigator’s estimates of probability of harm and benefits as judged by known facts or other available studies” [1]. Here ‘harms’, is substituted for risks, and ‘probability of risk’ has become ‘probability of harms’. With the language “as judged by known facts and other available studies” [1], the assumption seems to be that while the outcomes of the proposed research are unknown, there are some antecedent certainties relevant for assessing the proposed research. The language oscillates between certainty and probability but ultimately rests on an acceptance of determinism, that, according to Bursztajn, “makes experimentation a simple matter, just manipulate the variables and record what happens” [4]. Assuming that “one or more causes can be specified with certainty, experimenters can hold some possible causes constant in order to measure the effect others have” [4, pg 34]. That implies that causation itself is not subject to chance [4, pg 29 ]. Supporting that is the belief in the so-called crucial experiment [4 pg 25] that rests on two fundamental assumptions - that “the experimenter can account for and control all possible causal factors;” and that “the experimenter is assumed to be a detached observer in the sense that, aside from his planned manipulations, he does not influence the observed effects” [4 pg 26]. But both of these assumptions have become suspect in light of a probabilistic appreciation of science derived from Heisenberg’s principle of uncertainty. While someone might question the relevance of a principle of quantum physics to clinical research, there is substantial evidence that its implications for indeterminate causation have been appropriated in other disciplines, including the social sciences, economics, and philosophy [4 pg 55–56]. In contrast, medicine has remained wedded to Newtonian determinism [5].

What might account for this if not the persistent influence of Claude Bernard’s understanding of medicine as a science, grounded in a deterministic paradigm. “As a science, medicine necessarily has definite and precise laws which, like those of all the sciences, are derived from the criterion of experiment” [6]. This is science practiced under the principle of determinate causation since “ [f]or every observed effect the scientist seeks to isolate a specific cause or set of causes, as if it alone can account for the effect” [4 pg 24]. While not discounting use of probabilistic evidence, determinism so dominated Bernard’s thinking that it excluded causation as something subject to chance [7].

Contemporary clinical determinism is evident in a recent and representative discussion of risk/benefit ratio assessment. “The potential medical benefits to individual participants may outweigh the risks posed by the research interventions” [8]. While true, the opposite is also true, drawing attention to a relevant logical point. Truth is not relative to available evidence but our knowledge of truth is, otherwise there would be no discoverable truths [9 pg 95]. Here the need is to assess evidence for something true which at this point can only be assessed in probabilities.

Appreciating this requires a broader understanding of causation beyond cause as a ‘sufficient condition’ [9 pg 152-154]. If A is the cause of B, then A is understood to be a sufficient condition for B to occur. But causality shares other features more amenable to probability. One might say that A is the cause of B, inferring some predictability in their association, so that whenever A occurs, so does B, but assessed on the basis of some statistical invariance [9 pg 153]. In a clinical research context of many possibilities but where only one thing happens “under the rule of probability,” [10 pg 1] this broader appreciation of causation embraces the question critical under these conditions for science and ethics: how right must you be at this point [10 pg 2]? The answer lies in the application of statistics and probability theory which would, it is argued here, provide the indispensable justification for the conduct of clinical research [10].

Does this amount to reducing the ethics of clinical research to matters of statistics and probabilities? It does to the extent that both enable us to be rational about conducting clinical research in the face of its inherent uncertainty. Ethics, as a matter of reason, answers the question, what *should*, as distinct from *can*, one do? Aristotle observed that “every activity, artistic or scientific… has for its object the attainment of some good” [11 pg 25]. If the researcher concludes she should conduct the research, presumably she has identified the possibility of achieving some good. In the case of a Phase I trial, that would be to determine the safety of a novel drug. But since that will remain uncertain, the researcher has to proceed, justified on the probability that at least one of numerous acceptable possibilities will occur to that end.

Since this is as right as one can be under the circumstances, there are grounds for judging the research *as research* good, using the term descriptively in the way that one might judge a computer to be good. From there it is now possible to judge the research as good ethically. But this requires using ‘good’ prescriptively [12]. In ethics, the distinction between the descriptive and prescriptive uses of ‘good’ is critical. I might say of Ms. Jones that she is tall; and I might also say of her that she is good. Reasonably, it might appear that just as I am describing Ms. Jones as tall, I am also describing her as good. But if I use ‘good’ as an ethics term, I am saying that one ought to behave like Ms. Jones [13]. To use the term good prescriptively is to recognize a logical characteristic peculiar to the term itself that translates into a command – be like Ms. Jones! “To say something is good is to guide action” [12 pg 29]. Consequently, if the researcher accepts the ethical judgment of good, then she is saying, “I ought to do it.” But not, however, before making two additional determinations to secure objectivity. The first, that the judgment made of her particular research is universalizable, so that it would apply to all researchers finding themselves in a similar research situation. The second, that her judgment overrides alternative judgments of good relative to her research. That is to say, “A judgment is not moral if it does not provide, without further imperative premises, a reason for doing something” [12 pg 31]. In clinical research, since there is no certainty to justify doing it, justification, scientific and ethical, comes from probability. This is an essential consideration for deterministic medicine that it needs to take up seriously.

## Conclusion

Failure to do so will result in its continuing to ignore the probabilistic nature of clinical research, as well as misconstruing risk (probability) assessment which is indispensable for the science and ethics of the enterprise.
